# Promoter hypomethylation of *CDH7*: a novel epigenetic marker associated with cerebral small vessel disease

**DOI:** 10.3389/fgene.2026.1780415

**Published:** 2026-03-12

**Authors:** Jeeyeon Kim, Jihye Park, Keunsoo Kang, Young Ho Lee, Byoung-Soo Shin, Dae-Hyun Kim, Dong-Ick Shin, Seong Hwan Ahn, Jae Guk Kim, Hyun Goo Kang, Hyeseon Jeong, Kyu Sun Yum, Hee-Yun Chae, Do-Hyung Kim, Jei Kim

**Affiliations:** 1 Epigenetics Laboratory, Department of Neurology, College of Medicine and Hospital, Chungnam National University, Daejeon, Republic of Korea; 2 Department of Anatomy, College of Medicine, Chungnam National University, Daejeon, Republic of Korea; 3 Department of Microbiology, College of Bio-Convergence, Dankook University, Cheonan, Republic of Korea; 4 Department of Neurology, Research Institute of Clinical Medicine and Biomedical Research Institute, Medical School and Hospital, Jeonbuk National University, Jeonju, Republic of Korea; 5 Department of Neurology, Busan Regional Cardiocerebrovascular Disease Center, Dong-A University Hospital, Busan, Republic of Korea; 6 Department of Neurology, Chungbuk Regional Cardiocerebrovascular Disease Center, College of Medicine and Hospital, Chungbuk National University, Cheongju, Republic of Korea; 7 Department of Neurology, Chosun University Hospital, Gwangju, Republic of Korea; 8 Department of Neurology, Daejeon Eulji Medical Center, Eulji University School of Medicine, Daejeon, Republic of Korea; 9 Department of Neurology, Chungnam National University Hospital, Daejeon, Republic of Korea

**Keywords:** aging, CDH7, cerebral small vessel diseases, epigenetic marker, epigenome-wide association study, homocysteine, imaging features, promoter methylation

## Abstract

**Introduction:**

Cerebral small vessel disease (SVD), manifesting as white matter hyperintensities (WMH), lacunar infarctions, and cerebral microbleeds on magnetic resonance imaging (MRI), has been linked to developmental epigenetic alterations. This study aimed to identify and validate gene-specific promoter methylation changes as epigenetic markers associated with SVD, using MRI-defined imaging features and blood inflammatory cells.

**Methods:**

Genome-wide promoter methylation was profiled using the Infinium MethylationEPIC v2.0 array in peripheral inflammatory cells from 16 patients without SVD and 16 patients with all three imaging features, including WMH, lacunes, and microbleeds on MRI. Candidate CpGs were defined as consensus DMPs detected by both minfi and SeSAMe (nominal P < 0.05 in both pipelines with concordant direction), filtered by absolute delta beta >0.10 and promoter proximity (TSS200/TSS1500). Validation was performed to determine whether these gene-specific promoter methylations could serve as independent variables predicting the presence of SVD imaging features when combined with established cardiovascular risk factors, using data from 766 patients with ischemic stroke (53 [6.9%] without SVD and 713 [93.1%] with ≥1 SVD imaging feature). Hierarchical logistic regression analysis and a deep learning model were applied. Subgroup analyses using multinomial logistic regression were performed to assess whether gene-specific promoter methylation could independently predict WMH or lacunes.

**Results:**

EPIC profiling identified 17 promoter regions with significant differences between groups, corresponding to *CDH7, ZNF234, OR51A4, DEFB126, MAP3K8, TMCO6, TMEM191B, MMUT, TEX26, ZNF600, FAM240C, S100A13, S100A14, FLG2, MIR3667HG, RECK,* and *MIR662*. Among these, *CDH7*hypomethylation emerged as an independent predictor of any SVD imaging feature when combined with advanced age and hyperhomocysteinemia in both hierarchical logistic regression and deep learning analyses. Subgroup analysis demonstrated that *CDH7* hypomethylation independently predicted the presence of a isolated lacune, whereas no association was observed for isolated WMH.

**Conclusion:**

*CDH7* hypomethylation was identified and validated as an epigenetic marker predictive of MRI-defined SVD imaging features using blood inflammatory cells. This finding highlights the potential of epigenetic profiling for improving risk stratification in patients with cerebral SVD.

## 1 Introduction

Cerebral small vessel disease (SVD) is a common cerebrovascular condition characterized by neuroimaging features such as white matter hyperintensities (WMH), lacunes, and cerebral microbleeds on magnetic resonance imaging (MRI) (Pantoni, 2010). The burden of SVD is increasingly recognized as a major contributor to vascular cognitive impairment, intracerebral hemorrhage, and ischemic stroke (Wardlaw et al., 2013; Marini et al., 2020). Despite its clinical importance, the biological mechanisms underlying pathological changes in the small cerebral vessels remain incompletely understood.

Genetic susceptibility has been considered a key contributor to SVD. WMH, one of the principal imaging features of SVD, shows high heritability ranging from 54% to 80% in twin studies (Yang et al., 2023; Ten Kate et al., 2018). SVD is a heterogeneous condition encompassing both rare monogenic forms, such as cerebral autosomal dominant arteriopathy with subcortical infarcts and leukoencephalopathy (CADASIL) and cerebral autosomal recessive arteriopathy with subcortical infarcts and leukoencephalopathy (CARASIL), and more common polygenic or susceptibility variants (Marini et al., 2020). Genome-wide association studies (GWAS) have identified several genetic loci associated with SVD imaging features (Sargurupremraj et al., 2020; Malik et al., 2018). However, these variants explain only a limited proportion of the variance in WMH, estimated at approximately 29% (Sargurupremraj et al., 2020). This suggests that additional mechanisms beyond DNA sequence variation contribute to SVD development.

Epigenetic regulation represents a plausible biological link between genetic background, environmental exposures, and aging-related vascular injury. Hypertension is strongly associated with SVD, while diabetes, hypercholesterolemia, and smoking are also recognized risk factors (Hannawi, 2024; Wang et al., 2021). In addition, advancing age is one of the most important determinants of SVD, with imaging features observed in approximately 80% of individuals aged 65 years and in nearly all individuals aged 90 years (Haffner et al., 2016; Hilal et al., 2017). Chronic exposure to vascular risk factors and aging-related biological processes may induce epigenetic alterations that influence gene expression in pathways relevant to small vessel pathology (Yang et al., 2023).

Among epigenetic mechanisms, DNA methylation in gene promoter regions plays a critical role in regulating gene expression without altering the underlying DNA sequence (Jones and Baylin, 2002). Both aging (Heyn et al., 2012) and environmental influences (Marsit, 2015) are known to affect promoter methylation patterns. Therefore, profiling promoter methylation may help identify epigenetic markers that reflect the cumulative effects of vascular risk factors and aging in SVD. A previous epigenome-wide association study (EWAS) suggested that epigenetic alterations in immune response pathways and blood–brain barrier–related genes may be associated with WMH (Yang et al., 2023). Nevertheless, epigenetic markers underlying the broader spectrum of SVD imaging features—including lacunes and cerebral microbleeds—remain largely unexplored, limiting understanding of disease initiation and progression (Regenhardt et al., 2019; Yang et al., 2023).

Genome-wide investigations of genetic and epigenetic variation have advanced the study of complex diseases, but such approaches require large sample sizes and substantial resources to achieve robust statistical power and minimize false-positive findings (Pereira et al., 2023; Uffelmann et al., 2021; Campagna et al., 2021). In SVD research, additional methodological challenges arise from the high prevalence of imaging abnormalities in older adults. Because SVD features are present in most individuals over 65 years of age and the coexistence of multiple imaging features becomes increasingly common with advancing age (Haffner et al., 2016; Hilal et al., 2017), it is difficult to recruit truly unaffected controls or participants with isolated imaging phenotypes. These limitations complicate conventional case–control EWAS designs and may partly explain inconsistencies across previous studies.

To address these challenges, we adopted a phenotype-extreme design to enhance contrast in epigenetic profiles. This study aimed to profile and validate gene-specific promoter methylation changes associated with SVD imaging features on MRI using DNA derived from blood inflammatory cells. Given the difficulty in identifying older individuals without any SVD imaging features or with a single isolated feature, we compared participants without SVD imaging abnormalities to those exhibiting all three major imaging features (WMH, lacunes, and cerebral microbleeds). After identifying promoter methylation changes associated with the combined SVD phenotype, we further evaluated which individual imaging features were linked to the identified methylation markers.

## 2 Materials and methods

### 2.1 Patients and evaluation of traditional cardiovascular risk factors

To identify and validate gene-specific promoter methylation markers associated with cerebral SVD, buffy coats were obtained from 766 of 988 patients with ischemic stroke (mean age 71.4 ± 7.4 years; 476 men, 290 women) who were enrolled in a clinical trial titled “Multi-center, prospective, cohort study to evaluate the relationship of STroke Recurrence and Anti-PlatElet Resistance in ischemic stroke patients” (STRAPER study; ClinicalTrials.gov Identifier: NCT03823274; funding support: Yuhan Corporation, South Korea), and were older than 60 years (Table 1) (Kim et al., 2024). As part of the STRAPER study, all patients underwent brain MRI upon arrival at the emergency department of each participating hospital. MRI scanners included the following: Achieva 3.0 T (Philips, Amsterdam, Netherlands) at Chungnam National University and Jeonbuk National University Hospitals; Magnetom Avanto 1.5 T (Siemens, Erlangen, Germany) at Dong-A University Hospital; Ingenia Elition 3.0 T (Philips) at Chungbuk National University Hospital; Magnetom Skyra 3.0 T (Siemens) at Chosun University Hospital; and Magnetom Sonata 1.5 T (Siemens) at Daejeon Eulji Medical Center. MRI features of SVD—including lacunes (Lee et al., 2004), WMH (Fazekas et al., 1987; Wahlund et al., 2001), and cerebral microbleeds located in cortex and/or subcortex (Gregoire et al., 2009)—were assessed on T2-weighted imaging (T2WI), fluid-attenuated inversion recovery (FLAIR), gradient-recalled echo (GRE), and/or susceptibility-weighted imaging (SWI) sequences according to the previous criteria described in the STRAPER study protocol. From the STRAPER cohort, 16 patients with all three SVD imaging features (all-SVD group; mean age 67.5 ± 7.4 years; six men, 10 women), including WMH, lacune, and cerebral microbleed on MRI, were selected, along with 16 patients without any imaging feature (no-SVD group; mean age 62.2 ± 6.0 years, eight men, eight women). These two groups were used to profile and identify gene-specific promoter methylation alterations related to SVD. The identified alterations were subsequently validated in the entire cohort of 766 patients.

**TABLE 1:** Comparison of risk factors, blood tests, and *CDH7* methylation between patients with and without SVD.

Variables		No-SVD (n = 53)	Any-SVD (n = 713)	Total (n = 766)	*P*-value
Age (years, ±SD)		66.1 ± 5.7	71.8 ± 7.4	71.4 ± 7.4	<0.001
Sex (Women:Men, %)		16 (30.2%):37 (69.8%)	274 (38.4%):439 (61.6%)	290 (37.9%):476 (62.1%)	0.245
Risk factors	Hypertension (No:Yes, %)	26 (49.1%):27 (50.9%)	274 (38.4%):439 (61.6%)	300 (39.2%):466 (60.8%)	0.145
	Diabetes (No:Yes, %)	38 (71.7%):15 (28.3%)	485 (68.0%):228 (32.0%)	523 (68.3%):243 (31.7%)	0.648
	Smoking (No:Yes, %)	42 (79.2%):11 (20.8%)	563 (79.0%):150 (21.0%)	605 (79.0%):161 (21.0%)	1.000
Blood tests	GPT (U/L)	24.3 ± 19.4	23.5 ± 13.3	23.5 ± 13.8	0.655
	GOT (U/L)	27.9 ± 23.2	26.3 ± 12.4	26.4 ± 13.4	0.403
	Creatinine (mg/dL)	0.8 ± 0.2	0.8 ± 0.3	0.8 ± 0.3	0.216
	Homocysteine (μmol/L)	9.2 ± 2	11.6 ± 4.4	11.4 ± 4.3	<0.001
	Total cholesterol (mg/dL)	183.3 ± 43.8	179.7 ± 44	180 ± 44	0.563
	LDL (mg/dL)	108.7 ± 35.3	108.4 ± 38.6	108.4 ± 38.4	0.962
	HDL (mg/dL)	49.2 ± 11.6	46.8 ± 13.1	47 ± 13	0.189
	Triglyceride (mg/dL)	123.1 ± 78.8	140.2 ± 106.3	139 ± 104.7	0.251
	Hs-CRP (g/L)	2.3 ± 6.8	2.7 ± 7.1	2.7 ± 7.1	0.688
	White blood cell (/μL)	7.5 ± 2	7.6 ± 2.4	7.6 ± 2.4	0.879
	Hemoglobin (g/dL)	14.1 ± 1.4	13.7 ± 1.6	13.8 ± 1.6	0.167
	Platelet (103/μL)	237.7 ± 81.3	228.7 ± 57.8	229.3 ± 59.7	0.290
	Hemoglobin A1c (%)	6.4 ± 1.6	6.3 ± 1.2	6.3 ± 1.2	0.345
*CDH7* methylation (%)		26.0 ± 9.3	23.1 ± 9.7	23.3 ± 9.7	0.032

*CDH7*, cadherin-7; GOT, glutamic oxaloacetic transaminase; GPT, glutamic pyruvic transaminase; HDL, high-density lipoprotein; hsCRP, high-sensitivity C-reactive protein; LDL, low-density lipoprotein; SVD, cerebral small vessel disease.

Participants enrolled in the STRAPER study were evaluated for age, sex, and three cardiovascular risk factors: hypertension, diabetes mellitus, and smoking history. Additionally, 13 blood tests were performed, including glutamic pyruvic transaminase, glutamic oxaloacetic transaminase, creatinine, homocysteine, total cholesterol, low-density lipoprotein cholesterol, high-density lipoprotein cholesterol, triglyceride, high-sensitivity C-reactive protein, white blood cell count, hemoglobin, platelet count, and hemoglobin A1c. All samples were collected in the fasting state within 24 h of admission (Table 1). Patients with a diagnosis of atrial fibrillation, either before or after admission, were excluded from the present study in accordance with the STRAPER study criteria.

### 2.2 Blood collection and DNA extraction

For each participant in the STRAPER study, 3 mL of whole blood was collected in a citrate-coated tube. After centrifugation for 15 min at 100 × g, the buffy coat was separated and stored at −80 °C until further processing. DNA was extracted from buffy coats using the DNeasy Blood & Tissue Kit (Cat. no. 69506, Qiagen, Valencia, CA, United States) and stored at −20 °C until profiling and validation of gene-specific promoter methylation markers.

### 2.3 Inflammatory cell isolation from peripheral blood of ischemic stroke patients

Peripheral blood samples were collected from patients with ischemic stroke. To isolate T cells, B cells, and monocytes, buffy coats were first obtained by density gradient centrifugation using Ficoll (Ficoll-Paque PLUS, Cat. no. 17-1440-02, GE Healthcare). Subsequently, T cells (Dynabeads® CD3, Cat. no. 11151D, Invitrogen), B cells (Dynabeads® CD19 Pan B, Cat. no. 11143D, Invitrogen), and monocytes (Dynabeads® CD14, Cat. no. 11149D, Invitrogen) were isolated from the buffy coats using magnetic bead separation according to the manufacturer’s instructions. Isolated inflammatory cells were preserved in RNAlater (Cat. no. AM7020, Sigma-Aldrich) until DNA extraction. Genomic DNA was extracted using the DNeasy Blood & Tissue Kit (Qiagen) and stored at −20 °C until further use.

### 2.4 Profiling differentially methylated CpG sites in SVD

Methylation profiling was conducted using the Infinium MethylationEPIC array kit (v.2.0, Illumina Inc., San Diego, CA, United States) on DNA extracted from the buffy coats of the 16 no-SVD and 16 all-SVD patients selected from the STRAPER registry. Raw IDAT files covering 930,075 CpG sites were processed with minfi (Aryee et al., 2014) and SeSAMe (Poggiali et al., 2025) workflows. Minfi used Noob-based normalization and genome mapping, while SeSAMe applied Noob with dye-bias correction. Probes were retained if detection p value < 0.01 across all samples, probes overlapping common SNPs with MAF ≥0.01 were removed, and sex-chromosome probes were excluded, yielding 888,377 CpGs. Cell composition was estimated by reference-free deconvolution using RefFreeEWAS with K = 5 latent components (Houseman et. al., 2014), and SeSAMe used a blood reference from EpiDISH (Teschendorff et. al., 2017). K = 5 was selected to align with the five major leukocyte compartments used in the EpiDISH blood reference (B, NK, CD4T, CD8T, and neutrophils) and to keep the adjustment simple and stalbe for n = 32. Smaller K would merge distinct cell components, and larger K would add complexity and reduce degrees of freedom. Automatic covariate discovery screened metadata associated with top PCs with alpha 0.01 and up to 5 PCs, with safeguards against overfitting that capped covariates plus surrogate variables (SVs) at 25% of sample size. Surrogate variables showing strong association with case–control status were excluded (p value <1 × 10^−6^ or η^2^ > 0.5), where η^2^ denotes the proportion of variance in an SV explained by case–control status (Leek and Storey, 2007). Differential methylation was tested on M-values with a logit offset of 1e-4 using linear modeling with a test-control contrast and empirical Bayes moderation (Ritchie et al., 2015). Delta beta (Δβ) was defined as the difference in mean methylation beta values between test and control groups and used as the effect size criterion. Multiple testing used Benjamini–Hochberg false discovery rate (FDR), and significance was assessed at FDR <0.05 with |Δβ| > 0.1, though no CpG met this threshold in this dataset. Therefore, for candidate screening we defined consensus differentially methylated positions (DMPs) as CpGs with p value <0.05 in both pipelines and concordant direction, then, applied an effect-size filter of absolute delta beta >0.10 and promoter annotation limited to TSS200 or TSS1500 (Table 2; Supplementary Table S1). Variance partitioning was performed to quantify contributions of model features (Supplementary Table S2).

**TABLE 2:** Initial candidate genes with 19 CpG sites showing >10% methylation differences between no-SVD and all-SVD patients.

CG ID	Nearest gene	Chromosome	Distance to TSS (bp)	Mean Δβ (all-SVD − control)	*P*-value
cg12110659	*CDH7*	18	−416	0.1250	0.00302
cg23489630	*ZNF234*	19	−430	0.1165	0.00099
cg15962195	*OR51A4*	11	563	−0.1221	0.00180
cg24862298	*DEFB126*	20	−1170	0.1175	0.00286
cg18806716	*MAP3K8*	10	−431	0.1061	0.00354
cg27161585	*TMCO6*	5	−330	0.1031	0.00653
cg03311906	*TMEM191B*	22	−1264	−0.2202	0.00714
cg08259796	*MMUT*	6	831	−0.1004	0.00784
cg13614409	*TEX26*	13	−41	0.1287	0.00967
cg16848843	*ZNF600*	19	29	−0.1157	0.01191
cg00935887	*FAM240C*	2	−702	−0.1064	0.01208
cg02331910	*S100A13*	1	−571	−0.1080	0.02093
cg08477332	*S100A14*	1	771	−0.1629	0.02379
cg03439811	*FLG2*	1	1440	0.1351	0.03094
cg21714878	*MIR3667HG*	22	327	0.1057	0.03770
cg13392078	*RECK*	9	−388	−0.1185	0.04056
cg09066676	*MIR662*	16	−657	0.1033	0.04155
cg11343894	*S100A13*	1	−698	−0.1083	0.04319
cg02873163	*S100A13*	1	−573	−0.1092	0.04892

All-SVD, patients, patients with all SVD, imaging features including white matter hyperintensity, lacune, and microbleed on magnetic resonance imaging (MRI); Mean Δβ, mean methylation difference of a CpG site between no-SVD, and all-SVD, patients; No-SVD, patients, patients without SVD, imaging features on MRI; SVD, cerebral small vessel diseases; TSS, transcription start site.

### 2.5 Selection of target gene-specific promoter methylation

Target CpG sites were initially selected from the consensus DMP list (nominal P < 0.05 in both minfi and SeSAMe with concordant direction and |Δβ| > 0.10) and restricted to promoter-proximal annotations (TSS200/TSS1500). From these sites, secondary target CpG sites were chosen if located within promoter CpG islands, defined by a GC content >50% and an observed/expected ratio >0.6 in protein-coding genes, to support robust pyrosequencing design. For the final selection of target gene-specific promoter methylation for validation analysis, a methylation level >5% was used as an internal control (Kim et al., 2016) and as the threshold for positivity (Draht et al., 2016), consistent with previous pyrosequencing studies. After pyrosequencing of the secondary target gene-specific promoter methylations from the 16 no-SVD and all-SVD patients, genes showing >5% promoter methylation on pyrosequencing were selected as target genes for the validation study.

### 2.6 Gene-specific promoter methylation evaluation

**FIGURE 1:**
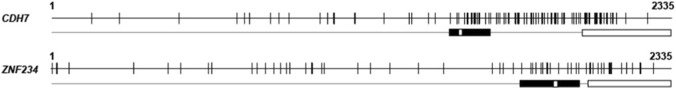
Genomic structure of promoter CpG islands and regions analyzed by bisulfite pyrosequencing for *CDH7* and *ZNF234* identified via the Illumina EPIC array. Promoter regions of *CDH7* (chr18:65,749,012-65,751,346) and *ZNF234* (chr19:44,139,554-44,141,888) were amplified and included exon 1 indicated by open boxes. Black boxes represent regions targeted in *CDH7* (177 bp) and *ZNF234* (223 bp) for bisulfite pyrosequencing, and the small open segment within each black box indicates the sequencing primer binding site for *CDH7* and *ZNF234*. Genomic coordinates are based on the GRCh38/hg38 reference genome.

Promoter methylation levels of *CDH7* (cadherin-7) and *ZNF234* (zinc finger protein 234) were evaluated using bisulfite pyrosequencing. Bisulfite conversion was performed on 1 μg of genomic DNA using a commercial kit (cat. no. D5002, Zymo Research, United States), and the converted DNA was stored at −20 °C until further analysis. For bisulfite pyrosequencing, we used the primer sets listed in Supplementary Table S3 to amplify regions within the promoter CpG islands of CDH7 (177 bp, chr18:65,749,012–65,751,346) and ZNF234 (223 bp, chr19:44,139,554–44,141,888) (Figure 1). Genomic coordinates are based on the GRCh38/hg38 reference genome. Polymerase chain reaction (PCR) for pyrosequencing was performed in a 20 μL reaction volume using a premix PCR kit (AccuPower® PyroHotStart Taq PCR PreMix, cat. no. K-2611, Bioneer, South Korea) with 1 μL (15 ng) of bisulfite-treated DNA and 0.1 mmol/L of each forward and reverse primer; one primer per gene was biotinylated at the 5′-end to amplify the target promoter region of each gene. PCR cycling conditions included denaturation at 95 °C for 5 min, followed by 45 cycles of 95 °C for 30 s, 55 °C for 30 s, and 72 °C for 30 s, with a final annealing/extension step at 72 °C for 10 min. Bisulfite pyrosequencing was then performed using the sequencing primer for individual genes, PyroMark Gold Q96 Reagents (cat. no. 972804, Qiagen), and a pyrosequencing machine (PyroMark Q96 ID, Qiagen). Methylation levels were calculated as the mean percentage methylation across all successfully analyzed CpG sites within each amplicon.

### 2.7 Cancer cell line preparation to evaluate the relationship of promoter methylation with expression of *CDH7*

To evaluate whether the *CDH7* promoter methylation related with the expression of the gene, we used eight cell lines including three lung cancer cell lines (H157, H460, and H1299), one colon cancer cell line (HCT116) and four renal cancer cell lines (A704, SNU349, SNU482, and SNU1272). DNA and RNA from the cell lines were extracted using a DNA/RNA extraction kit (AllPrep DNA/RNA Mini Kit, Cat. No. 80204, Qiagen) following the manufacturer’s instructions, and, DNA stored at −20 °C and RNA stored at −80 °C until next use.

### 2.8 Real-time RT-PCR

Two μg of total RNA from each gene was transcribed using a reverse transcription kit (AccuPower® RT PreMix, Cat. No.; K2041, Bioneer, South Korea) according to the manufacturer’s instructions. To quantitatively evaluate the expression of *CDH7*, real-time RT-PCR was performed (Step-One Real-Time PCR systems, Applied Biosystems) using 20x CDH7 probe (Taqman® Gene Expression Assays, Cat. No. Hs00917727_m1, Applied Biosystems, ThermoFisher Scientific, CA, United States) For an internal control, we used beta actin (*ACTB*, Taqman® Gene Expression Assays, Hs01060665_g1, ThermoFisher Scientific) expression. The reaction mixture for real time RT-PCR contained 10 μL of TaqMan® Universal Master Mix (Cat. No.; 4369016, ThermoFisher Scientific), 1 μL of 20x primer probe mix, 7 μL of distilled water and 2 μL of cDNA. Amplification was performed with the following steps: 10 min at 95 °C and 40 cycles (95 °C, 30 s, 60 °C, 30 s, 72 °C, 30 s). The real-time RT-PCR reactions for each gene were done in duplicate.

To analyze the real-time RT-PCR results, the average cycle number (*C*_*T*_) of the reaction when it crossed a threshold value was determined for each reaction. The differences in *C*_*T*_ (Δ*C*_*T*_) between *CDH7* and the reference gene *ACTB* were calculated by subtracting *C*_*T*_ of *ACTB* from *C*_*T*_ of *CDH7* to normalize expression levels.

### 2.9 Relationship analysis of *CDH7* methylation with expression in non-cancer human blood and brain tissues

To evaluate whether the relationship between *CDH7* methylation and gene expression extends beyond cancer-derived models, we performed a secondary analysis of publicly available GEO datasets containing matched DNA methylation and gene expression microarray data from non-cancer human blood and brain tissues. Four datasets met the inclusion criteria: GSE117931 (GPL13534 methylation and GPL14951 expression), GSE49065 (GPL13534 and GPL11532), GSE15745 (GPL8490 and GPL6104), and GSE38609 (GPL8490 and GPL10558). Sample matching was performed within each dataset using shared sample identifiers in the GEO metadata. *CDH7* expression was summarized as the mean signal across *CDH7*-mapped expression probes for each platform, and *CDH7* promoter methylation was summarized as the mean beta value across promoter-associated *CDH7* CpG probes (450K: *CDH7* probes annotated to TSS1500/TSS200/5′UTR/1stExon; 27K: *CDH7* probes within ±1,500 bp of the transcription start site). Associations between methylation and expression were evaluated using Spearman rank correlation separately for each dataset.

### 2.10 Statistical and deep learning model analysis

The dataset included *CDH7* methylation data together with three clinical cardiovascular risk factors and 13 blood test variables from 766 STRAPER registry patients for the validation analysis (Table 1). After confirming dataset completeness, the final dataset was prepared by imputing missing values using the missForest method (Stekhoven and Bühlmann, 2012) from the missCompare imputation algorithm package developed for R (version 4.5.1) as previously described (https://github.com/Tirgit/missCompare).

Using the complete dataset, differences in cardiovascular risk factors, blood test results, and *CDH7* promoter methylation levels were compared between no-SVD and any-SVD patients, who had ≥1 WMH, lacune, and/or microbleed SVD imaging features. An independent t-test was applied to continuous variables, and a chi-squared test was applied to categorical variables. Differences in *CDH7* methylation levels among monocytes, T cells, and B cells were assessed using analysis of variance (ANOVA). Correlation analyses were performed to evaluate the relationship between *CDH7* promoter demethylation and gene expression levels.

Hierarchical logistic regression analysis was then performed to evaluate whether the prediction of any-SVD based on traditional cardiovascular risk factors and blood tests could be enhanced by adding *CDH7* methylation levels. Model performance was assessed using the area under the receiver operating characteristic curve (AUC).

*CDH7* hypomethylation was also evaluated as a potential predictor of any-SVD in combination with clinical cardiovascular risk factors and blood tests using deep learning model algorithms implemented with PyTorch and scikit-learn. A residual multilayer perceptron (MLP) architecture with learnable per-feature gates was constructed. Training of the deep learning model used logit-based binary focal loss with optimization of the weighting factor (α) and focusing parameter (γ) to address class imbalance in the dataset. To mitigate overfitting, five-fold stratified cross-validation was performed, and Optuna’s Tree-structured Parzen Estimator (TPE) sampler was used to optimize model hyperparameters. For cross-validation, the validation dataset was divided into two subsets: 70% for training and 30% for testing. Five-fold cross-validation of the logistic regression model was performed on the 70% training dataset, and prediction performance was validated using the 30% test dataset. The deep learning model was evaluated using AUC, accuracy, and the Matthews correlation coefficient (MCC) (Chicco and Jurman, 2020). Global interpretability of the any-SVD prediction model was assessed with KernelSHAP applied to a random background across folds and repeats. Mean absolute Shapley Additive exPlanations (SHAP) values were calculated across all validation samples, folds, and experimental repeats to establish feature importance.

The Optuna-selected configuration used three residual hidden layers (37-64-128 units) with ReLU activations, batch normalization, and dropout (base rate 0.63, decayed by layer), followed by a 16-unit classifier head. Models were optimized with Adam (learning rate 0.0021, weight decay 0.0051), batch size 32, up to 99 epochs with early stopping, and focal loss parameters alpha = 0.17 and gamma = 1.11.

Finally, *CDH7* hypomethylation was assessed as a predictor of specific SVD imaging features using multinomial logistic regression analysis. Subgroups with WMH, lacune, or microbleed were separately identified from the total validation dataset. Among 766 patients, the WMH subgroup (n = 638) included patients with isolated WMH (n = 221), who had only WMH without coexisting lacune or microbleed, and patients with coexistence-WMH (n = 462), who had WMH with concurrent lacune and/or microbleed imaging features on MRI. The lacune subgroup (n = 406) included patients with isolated lacune (n = 24), who had only lacune without coexisting WMH or microbleed, and patients with coexistence-lacune (n = 382), who had lacune with concurrent WMH and/or microbleed imaging features on MRI. Multinomial logistic regression analysis was performed for the WMH subgroup by comparing isolated WMH and coexistence-WMH with no-SVD patients, and for the lacune subgroup by comparing isolated lacune and coexistence-lacune with no-SVD patients, using the cardiovascular risk factors, blood tests, and *CDH7* methylation data. *CDH7* hypomethylation was further evaluated as an independent predictor of the isolated WMH and isolated lacune imaging features. The microbleed-only imaging feature was observed in only five patients from the validation dataset, which precluded multinomial logistic regression analysis for this group. All statistical analyses were performed using SPSS (version 29.0, IBM Corp., United States) and R packages (version 4.5.1). Statistical significance was set at *p* < 0.05.

### 2.11 Ethical considerations

The protocol of the present study for the retrospective use of buffy coat DNA from STRAPER participants was reviewed and approved by the Institutional Review Board (IRB) of Chungnam National University Hospital (CNUH IRB 2023-10-025). The collection and use of buffy coat samples in the STRAPER study were approved prior to study initiation by the Institutional Review Boards of six participating hospitals in South Korea: Chungnam National University Hospital, Daejeon (CNUH-2018-12-033); Jeonbuk National University Hospital, Jeonju (CUH-2019-01-027); Dong-A University Hospital, Busan (DAUHIRB-19-048); Chungbuk National University Hospital, Cheongju (CBNUH-2019-01-015); Chosun University Hospital, Gwangju (CHOSUN-2019-02-006); and Daejeon Eulji Medical Center (EMC-2019-01-009-001). The study was conducted in accordance with the Declaration of Helsinki. Written informed consent was obtained from all participants prior to enrollment in the STRAPER study.

## 3 Results

**FIGURE 2:**
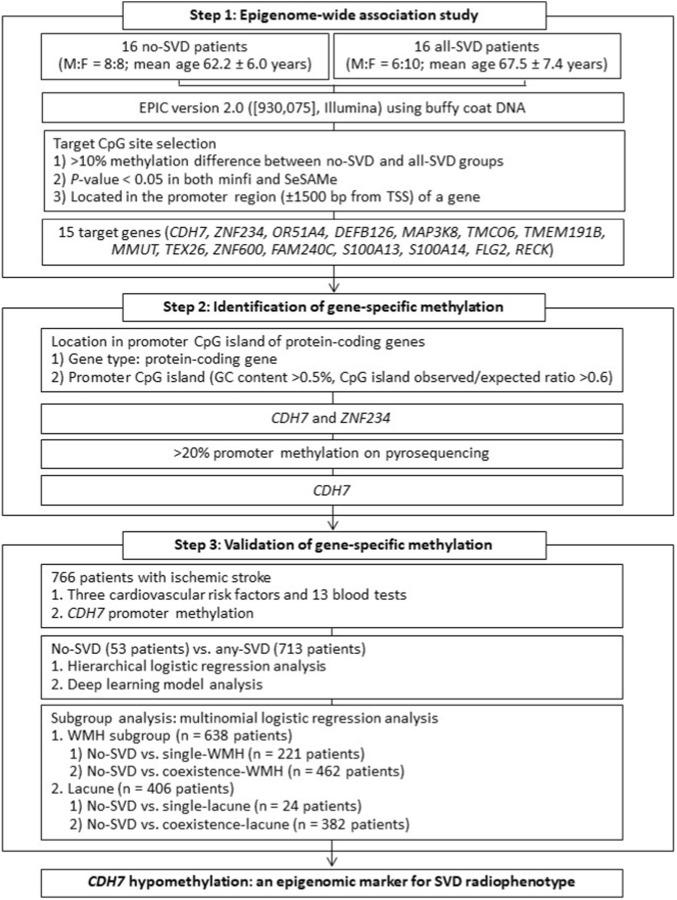
Flow diagram for profiling and validating gene-specific promoter methylation changes associated with cerebral small vessel disease (SVD) imaging features. All-SVD: patients presenting with all SVD imaging features; Any-SVD: patients presenting with ≥1 SVD imaging feature; F: female; M: male; SVD: cerebral small vessel disease imaging features, including white matter hyperintensity (WMH), lacune, and microbleed detected on magnetic resonance imaging; TSS: transcription start site.

The present study was conducted in three steps: (1) EWAS using blood inflammatory cells from patients with SVD; (2) identification of target gene-specific promoter methylation CpG sites associated with SVD imaging features; and (3) validation of the target gene-specific promoter methylation changes as epigenetic markers (Figure 2).

### 3.1 Profile and identification of gene-specific promoter methylation changes associated with SVD imaging features

**FIGURE 3:**
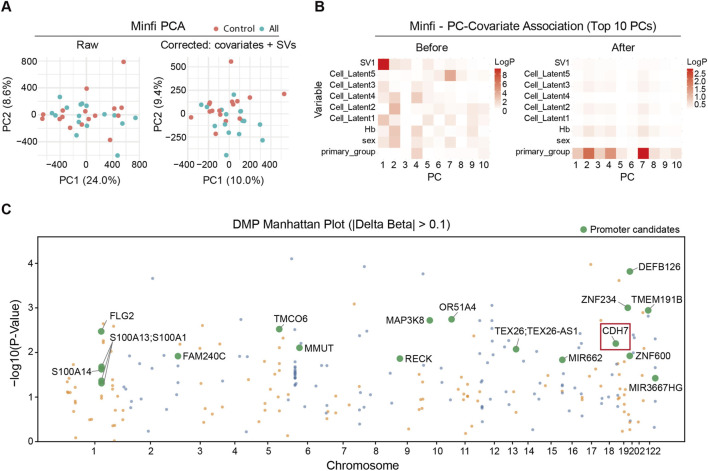
Covariate correction and promoter-candidate screening in the EPICv2 epigenome wide association study (EWAS) cohort. **(A)** Principal component analysis (PCA) of minfi beta values for control and all-cerebral small vessel diseases (SVD) group samples before and after covariate and surrogate variable (SV) adjustment. **(B)** Principal component (PC)–covariate association heatmaps for the top 10 PCs before and after correction, showing reduced associations with sex, hemoglobin (Hb), and cell-composition covariates while preserving primary group signal. **(C)** Manhattan plot of CpG sites with |Δβ| > 0.10, highlighting promoter-proximal candidates that meet P < 0.05 in both minfi and SeSAMe and are labeled by gene.

During the first EWAS step, the methylation status of 930,075 genome-wide CpG sites was evaluated using the MethylationEPIC v2 array with buffy coat DNA from 16 no-SVD and 16 all-SVD imaging feature patients. We analyzed raw IDAT files using IlluMeta (https://github.com/kangk1204/illumeta), which utilized both minfi and SeSAMe pipelines with Noob-based normalization, followed by an optimal covariate adjustment with sex, Hb, cell composition estimates, and one surrogate variable. This adjustment was evaluated with principal component analysis (PCA) plots (Figure 3A) and PC covariate association heatmaps. The principal component (PC) covariate association heatmaps clearly indicated attenuation of covariate-driven structure while retaining the primary group signal (Figure 3B). Variance partitioning showed that cell-composition terms explained most of the methylation variance (minfi latent components 0.764 and SeSAMe leukocyte fractions 0.554), whereas the primary group explained only up to 0.019, with sex, Hb, SV1, and residuals accounting for the remainder (Supplementary Table S2). These results indicated that cell-type heterogeneity was the dominant source of methylation variability in this blood-based EWAS, underscoring the necessity of cell-composition adjustment.

Genomic inflation factors were close to 1 for all pipelines, with lambda 1.07 for minfi and 0.98 for SeSAMe, indicating appropriate calibration and no substantial test statistic inflation. Despite these careful adjustments, no CpG sites passed FDR <0.05. Given the blood-based design with high cell-type heterogeneity and the small sample size, we set a screening threshold of p value <0.05 together with an effect-size filter of |Δβ| > 0.10 and promoter proximity (TSS200/TSS1500). To increase robustness, candidates were restricted to the intersection of minfi and SeSAMe results with concordant direction. These intersection CpGs were defined as consensus DMPs. The manhattan plot highlights these promoter proximal candidates among CpG sites with absolute Δβ greater than 0.10 (Figure 3C). This yielded 19 CpG sites mapping to 17 genes including *CDH7, ZNF234, OR51A4, DEFB126, MAP3K8, TMCO6, TMEM191B, MMUT, TEX26, ZNF600, FAM240C, S100A13, S100A14, FLG2, MIR3667HG, RECK,* and *MIR662* (Table 2; Supplementary Table S1). Among these, *CDH7* (cg12110659) and *ZNF234* (cg23489630) showed consistent promoter region hypermethylation in the all-SVD group with delta beta around 0.12 in both pipelines and were prioritized for downstream validation.

Genomic inflation factors were close to 1 for all pipelines, with lambda 1.07 for minfi and 0.98 for SeSAMe, indicating appropriate calibration and no substantial test statistic inflation. Despite these careful adjustments, no CpG sites passed FDR <0.05. Given the blood-based design with high cell-type heterogeneity and the small sample size, we set a screening threshold of *p* value <0.05 together with an effect-size filter of |Δβ| > 0.10 and promoter proximity within 1500 bp upstream or downstream of the transcription start site (TSS). To increase robustness, candidates were restricted to the intersection of minfi and SeSAMe results with concordant direction. These intersection CpGs were defined as consensus DMPs. The manhattan plot highlights these promoter proximal candidates among CpG sites with absolute Δβ greater than 0.10 (Figure 3C). This yielded 19 CpG sites mapping to 17 genes including *CDH7, ZNF234, OR51A4, DEFB126, MAP3K8, TMCO6, TMEM191B, MMUT, TEX26, ZNF600, FAM240C, S100A13, S100A14, FLG2, MIR3667HG, RECK,* and *MIR662* (Table 2; Supplementary Table S1). Among these, *CDH7* (cg12110659) and *ZNF234* (cg23489630) showed consistent promoter region hypermethylation in the all-SVD group with delta beta around 0.12 in both pipelines and were prioritized for downstream validation.

Evaluation of promoter methylation status revealed that *CDH7* pyrosequencing demonstrated >20% methylation (no-SVD: 23.1%; all-SVD: 25.8%), whereas *ZNF234* pyrosequencing showed ≤5% methylation (no-SVD: 2.8%; all-SVD: 5.8%) in the 16 no-SVD and 16 all-SVD patients selected for the MethylationEPIC array (Supplementary Table S4). *ZNF234* showed approximately 5% methylation at the internal control and at the positivity threshold of pyrosequencing in the CpG sites analyzed. Consequently, *CDH7* demonstrating >20% promoter methylation was finally selected as the potential blood-based DNA methylation biomarkers for subsequent validation experiments.

### 3.2 Validation of *CDH7* hypomethylation as an independent variable to predict SVD imaging feature

#### 3.2.1 Preparation of the validation dataset

For the third step, which involved validation of *CDH7* promoter methylation as an independent variable associated with SVD imaging features, a complete dataset was prepared. This dataset included 19 variables (age, sex, three cardiovascular risk factors, 13 blood tests, and *CDH7* methylation) for 766 patients enrolled in the present study. Among the 14,554 total values, 564 missing values (3.8%) were imputed using the missForest method (Supplementary Table S5).

#### 3.2.2 Frequency of the isolated and coexisting SVD imaging features in the total patient cohort

**FIGURE 4:**
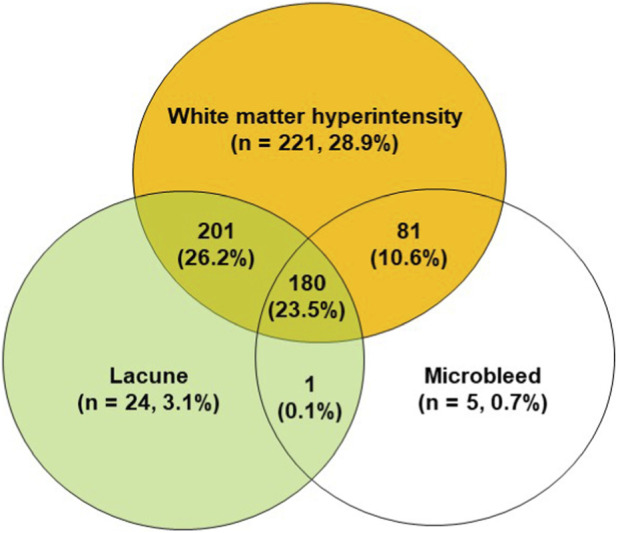
Distribution of patients with isolated or coexisting imaging features of cerebral small vessel diseases in the validation dataset.

The validation dataset included 766 patients, among whom 53 patients (6.9%) exhibited no cerebral SVD imaging features on MRI, whereas 713 patients (93.1%) demonstrated at least one imaging feature, including WMH, lacune, or microbleed (Figure 4). One-third of the any-SVD patients (n = 250, 32.6% of the total cohort) demonstrated a isolated imaging feature, consisting of isolated WMH (n = 221, 28.9%), isolated lacune (n = 24, 3.1%), or isolated microbleed (n = 5, 0.7%) (Figure 4). Two-thirds of the any-SVD patients (n = 545; 71.1%) simultaneously exhibited two or more imaging features, including combined lacune and WMH (n = 201, 26.2%), WMH and microbleed (n = 81, 10.6%), lacune and microbleed (n = 1, 0.1%), or all three imaging features of WMH, lacune, and microbleed (n = 180, 23.5%) on MRI (Figure 4).

#### 3.2.3 Comparison of *CDH7* promoter methylation among peripheral inflammatory cell types

**FIGURE 5:**
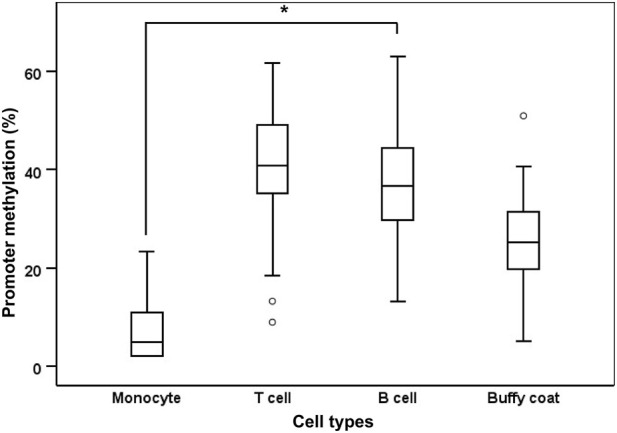
Box plots showing the promoter methylation status of *CDH7* in monocytes, T cells, B cells, and buffy coats from the peripheral blood of 89 patients with ischemic stroke. Within each box, horizontal white lines indicate median values; boxes represent the interquartile range (25th–75th percentiles); whiskers denote adjacent values; and dots represent outliers. *P < 0.01 by analysis of variance (ANOVA) comparing methylation levels among monocytes, T cells, and B cells.

To assess differences in *CDH7* promoter methylation among peripheral inflammatory cell types comprising the buffy coat, we isolated monocytes, T cells, and B cells from the peripheral blood of 89 patients (mean age, 64.4 ± 3.5 years; men/women = 56/33) out of 766 participants in the STRAPER cohort. Comparison across cell types revealed that monocytes exhibited significantly lower *CDH7* methylation levels (monocytes, 6.95% ± 5.46%; T cells, 41.04% ± 10.23%; B cells, 36.79% ± 10.80%; *P* < 0.001) than either T or B cells (Figure 5). The mean *CDH7* methylation level in buffy coats (26.10% ± 9.80%) approximated the average methylation across the three inflammatory cell types (Figure 5).

#### 3.2.4 Relationship between *CDH7* promoter methylation and gene expression

**FIGURE 6:**
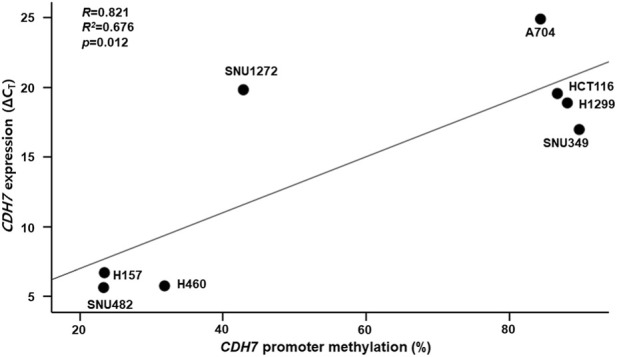
Relationship between CDH7 promoter methylation and gene expression across eight human cancer cell lines exhibiting different levels of promoter methylation. The ΔCt value represents the difference in threshold cycles between *CDH7* and the internal control beta actin (*ACTB)*, calculated by subtracting C_T_ of *ACTB* from C_T_ of *CDH7*.

To examine whether *CDH7* promoter methylation was associated with gene expression, we analyzed the relationship between *CDH7* methylation levels and ΔCt values (*CDH7* normalized to *ACTB*) across eight human cancer cell lines. Correlation analysis revealed that ΔCt values increased with higher levels of *CDH7* promoter methylation (R = 0.821, R^2^ = 0.676, *P* = 0.012; Figure 6), indicating that *CDH7* expression decreased as promoter methylation increased.

In additional matched-sample analyses of non-cancer public datasets (Supplementary Figure S1), inverse but non-significant associations were observed in blood datasets (GSE117931: rho = −0.194, P = 0.249; GSE49065: rho = −0.156, P = 0.510). Dataset-level associations in brain tissues were also non-significant (GSE15745: rho = −0.060, P = 0.187; GSE38609: rho = 0.137, P = 0.514). In an exploratory region-level analysis of GSE15745, the cerebellum showed a modest inverse association (rho = −0.217, P = 0.018), whereas other regions were not significant.

#### 3.2.5 Comparison of prediction performances before and after adding *CDH7* methylation to the cardiovascular risk factors and blood tests

In the comparison of cardiovascular risk factors and blood tests, any-SVD patients were significantly older (any-SVD = 71.8 ± 7.4 years; all-SVD = 66.1 ± 5.7 years, *p* < 0.001) and had higher homocysteine levels (any-SVD = 9.2 ± 2.0 μmol/L; all-SVD = 11.6 ± 4.4 μmol/L, *p* < 0.001) compared to no-SVD patients (Table 1). The methylation level of *CDH7* was lower in any-SVD patients (23.1% ± 9.7%) than in no-SVD patients (26.0% ± 9.3%, *p* = 0.032) (Table 1).

Hierarchical logistic regression analysis was conducted to evaluate whether adding *CDH7* methylation to cardiovascular risk factors and blood tests enhanced prediction performance for the presence of any-SVD imaging feature. Logistic regression analysis performed with cardiovascular risk factors and blood tests identified older age and higher homocysteine levels as independent variables for predicting any-SVD imaging feature (Table 3). When *CDH7* methylation level was added to cardiovascular risk factors and blood tests, *CDH7* hypomethylation was included as an additional independent variable alongside older age and higher homocysteine levels for predicting the presence of any-SVD imaging feature. AUC of the prediction models improved from 0.819 to 0.833 after *CDH7* methylation was added to other variables (Table 3).

**TABLE 3:** Comparison of predictive performance before and after adding *CDH7* methylation to cardiovascular risk factors and blood tests.

Multivariable logistic regression	ROC analysis	Risk factors + blood tests			Risk factors + blood tests + *CDH7* methylation		
		B	Exp(B)	*P*-value	B	Exp(B)	*P*-value
Variables	Constant	7.397	1630.317	0.011	6.911	1003.215	0.019
	Age	0.135	0.874	<0.001	0.142	0.867	<0.001
	Homocysteine	−0.203	0.816	<0.001	−0.199	0.82	<0.001
	*CDH7* methylation	​	​	​	0.04	1.041	0.013
ROC analysis	AUC (95% CI)	0.819 (0.773–0.866)			0.833 (0.789–0.878)		
	P-value	<0.001			<0.001		

AUC, the area under the receiver operating characteristic curve; B, coefficient; *CDH7*, cadherin-7; CI, confidence interval; Exp(B), odds ration; ROC, receiver operating characteristic.

#### 3.2.6 Deep learning model analysis to predict any-SVD imaging features

**FIGURE 7:**
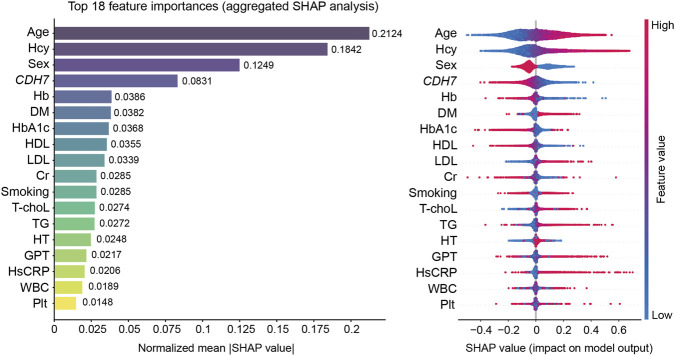
Feature importance (left panel) and impact on model output (right panel) of individual variables derived from aggregated Shapley Additive exPlanations (SHAP) analysis. CDH7, cadherin-7 promoter methylation; DM, previous history of diabetes; GPT, glutamic pyruvic transaminase; Hb, hemoglobin; HbA1c, hemoglobin A1c; Hcy, homocysteine; HDL, high-density lipoprotein; hsCRP, high-sensitivity C-reactive protein; HT, previous history of hypertension; LDL, low-density lipoprotein; Plt, platelet; TG, triglyceride; T-chol., total cholesterol; WBC, white blood cell.

Deep learning model analysis was conducted to evaluate whether *CDH7* methylation could serve as an important variable for predicting the presence of any-SVD imaging features when included alongside cardiovascular risk factors and blood tests. Similar to the logistic prediction model, *CDH7* hypomethylation ranked among the top four features on the aggregated SHAP analysis, together with older age, elevated homocysteine levels, and male sex, for the prediction of any-SVD imaging features (Figure 7). Notably, sex was not retained as an independent variable in the logistic regression analysis. The deep learning prediction model demonstrated aggregated performance with an AUC of 0.783 (95% confidence interval: 0.777–0.789), an accuracy of 0.878 (0.866–0.899), and a MCC of 0.395 (0.387–0.403).

#### 3.2.7 Comparison of prediction performances by individual SVD imaging features

Evaluation of *CDH7* hypomethylation as an independent predictor for specific SVD imaging features revealed differential contributions. Multinomial logistic regression analysis of the lacune subgroup identified *CDH7* hypomethylation and elevated homocysteine as independent variables for predicting the isolated lacune imaging feature. For the coexistence-lacune imaging feature, age was incorporated into the model along with *CDH7* hypomethylation and elevated homocysteine (Table 4). Conversely, multinomial logistic regression analysis of the WMH subgroup did not identify *CDH7* hypomethylation as a variable in the prediction model for isolated WMH. However, *CDH7* hypomethylation emerged as an independent variable in the prediction model for the coexistence-WMH imaging feature (Table 4). Analysis of the microbleed subgroup was not performed due to the small sample size, as only five patients demonstrated an isolated microbleed imaging feature. These findings suggest that *CDH7* hypomethylation functions as an independent marker for predicting the presence of isolated lacune imaging features rather than isolated WMH imaging features.

**TABLE 4:** Comparison of predictive performance between isolated and coexisting SVD imaging features.

No-SVD vs. WMH							No-SVD vs. Lacune					
Isolated WMH				Coexistence WMH			Isolated lacune			Coexistence lacune		
Variables	B	Exp(B)	*P*-value	B	Exp(B)	*P*-value	B	Exp(B)	*P*-value	B	Exp(B)	*P*-value
Constant	−8.146	​	0.014	−7.32	​	0.022	0.617	​	0.911	−8.584	​	0.014
Age	0.14	1.151	<0.001	0.153	1.165	<0.001	​	​	​	0.156	1.168	<0.001
Homocysteine	0.182	1.2	0.003	0.213	1.237	<0.001	0.259	1.295	0.002	0.268	1.307	<0.001
*CDH7* methylation	​	​	​	−0.044	0.957	0.008	−0.06	0.942	0.042	−0.045	0.956	0.012

B, coefficient; Exp(B), odds ration; SVD, small vessel disease; *CDH7*; cadherin-7, WMH, white matter hyperintensity.

## 4 Discussion

This study identified and validated promoter hypomethylation of *CDH7* as an independent epigenetic marker associated with cerebral small vessel disease (SVD) imaging features. The association was particularly evident when *CDH7* hypomethylation was considered alongside advancing age and elevated homocysteine, both established vascular risk factors. Notably, *CDH7* hypomethylation showed a stronger relationship with lacunes than with WMH, while assessment of microbleeds was limited by the small number of patients with an isolated microbleed phenotype. These findings suggest that *CDH7* promoter hypomethylation may represent a phenotype-specific epigenetic marker, especially for lacunar pathology.

Because direct sampling of brain tissue is rarely feasible (Husby, 2020), increasing attention has been directed toward identifying peripheral biomarkers that may reflect disease-related epigenetic alterations. Buffy coats, composed largely of circulating inflammatory cells, offer an accessible surrogate tissue for exploring systemic epigenetic mechanisms (Gunasekara et al., 2019). Nevertheless, DNA methylation profiles differ substantially among immune cell subtypes, reflecting both their developmental origins (Roadmap E pigenomics Consortium et al., 2015) and disease-associated influences (Schultz et al., 2015). In this context, our finding that *CDH7* methylation levels were lower in monocytes than in T or B cells suggests that cell type–specific epigenetic variability may partly underlie the overall hypomethylation signal observed in buffy coat samples. Thus, *CDH7* methylation measured in peripheral blood is likely to represent a composite epigenetic signature shaped by both shifts in immune cell composition and cell-intrinsic methylation alterations. This interpretation is in line with previous reports demonstrating cell type–dependent methylation differences in genes implicated in atherosclerosis (Kim et al., 2023), and supports the notion that epigenetic heterogeneity among inflammatory cell subsets may be linked to vascular pathological processes. While these observations support the potential utility of buffy coats as a source of epigenetic biomarkers, further studies using purified immune cell populations and longitudinal sampling will be important to disentangle cell-specific contributions and to better understand their functional and clinical relevance.

To explore the potential functional relevance of *CDH7* methylation, we first examined its association with gene expression across multiple human cancer cell lines. Higher levels of promoter methylation were associated with lower *CDH7* expression, consistent with a potential regulatory role of promoter methylation. Analyses of non-cancer human blood and brain tissues from publicly available datasets also suggested an inverse relationship between *CDH7* promoter methylation and gene expression; however, these associations were generally modest or statistically non-significant. Nevertheless, the overall direction of the associations observed in both cancer cell lines and independent public datasets supports a potential relationship between *CDH7* promoter methylation and gene expression. However, the observed associations in non-cancer tissues and cancer cell lines may not fully reflect regulatory mechanisms in normal vascular or neural tissues that are directly relevant to cerebral small vessel disease (SVD). Further studies using non-cancer human brain and endothelial samples are needed to determine whether *CDH7* promoter methylation contributes to gene regulation in the pathogenesis of SVD.

The validation dataset had inherent limitations, as only 7% of patients exhibited no SVD imaging features, resulting in a pronounced class imbalance. Such imbalance can affect model performance by inflating overall accuracy while reducing sensitivity to the minority class. To minimize this effect, we applied focal loss during deep learning training, used stratified cross-validation, and reported the Matthews correlation coefficient (MCC), which is less influenced by skewed class distributions. Nevertheless, residual bias related to the small no-SVD subgroup cannot be fully excluded, and model performance may differ in populations with a higher proportion of individuals without SVD. Subsequent validation of the identified markers was conducted in 766 patients with ischemic stroke presenting with various combinations of WMH, lacune, and/or microbleed imaging features on MRI. Validation analyses included hierarchical logistic regression and deep learning models. In addition, multinomial logistic regression was performed to evaluate whether CDH7 hypomethylation independently predicted the presence of specific SVD imaging features. Although this study did not adopt a longitudinal or large-scale case–control design, this multi-step analytical framework represents a pragmatic strategy for identifying epigenetic markers associated with SVD imaging phenotypes in aging populations, where SVD-free individuals are relatively uncommon and mixed imaging features frequently coexist.

The preferential association between *CDH7* hypomethylation and lacunes, but not WMH, supports the concept that different SVD imaging phenotypes arise from partially distinct biological mechanisms. WMH is typically linked to chronic hypoperfusion, demyelination, and gliosis (Gouw et al., 2011), whereas lacunes reflect cavitated tissue loss following small subcortical infarcts and are more directly related to structural small vessel pathology (Gouw et al., 2011). This raises the possibility that altered *CDH7* methylation may influence endothelial adhesion or vascular wall stability, thereby predisposing small penetrating arteries to occlusive injury rather than diffuse white matter damage. Although speculative, this interpretation aligns with the stronger vascular structural component underlying lacunar infarction.

Microbleeds represent an additional layer of heterogeneity, as lobar microbleeds are often associated with cerebral amyloid angiopathy, whereas deep microbleeds are more closely linked to hypertensive arteriopathy (Zhu et al., 2023; Rodrigues et al., 2018). We did not classify microbleeds by anatomical location, which may have obscured phenotype-specific associations. Future studies incorporating detailed topographical analyses and longitudinal follow-up will be necessary to determine whether *CDH7* methylation contributes to hemorrhagic small vessel injury and whether its effects differ across microangiopathy subtypes.

*CDH7* belongs to the cadherin superfamily (Paulson et al., 2014), which is central to cell–cell adhesion and intracellular signaling (Maître and Heisenberg, 2013). Cadherin-related pathways, including those involving vascular endothelial cadherins, are essential for endothelial integrity and blood–brain barrier stability (Wang et al., 2018). Disruption of these pathways has been implicated in vascular cognitive impairment and neurodegeneration (Andjelkovic et al., 2023). Although *CDH7* itself has been studied mainly in neural development (Kuwako et al., 2014; Nakagawa and Takeichi, 1998) and neuropsychiatric disease (Sklar et al., 2008), emerging pathway analyses link it to Rho signaling and cytoskeletal regulation (Jickling et al., 2022), processes relevant to vascular remodeling and endothelial function (Jickling et al., 2022). Together with prior reports implicating endothelial dysfunction, inflammation, and extracellular matrix remodeling in SVD progression, our findings suggest that *CDH7* hypomethylation may contribute to small vessel pathology through mechanisms involving vascular stability.

Hyperhomocysteinemia was identified as an independent variable in the any-SVD prediction model in the present study. This condition is an established independent risk factor for cardiovascular disease, stroke, and dementia (Mandaviya et al., 2014; Ganguly and Alam, 2015). Elevated homocysteine levels are known to increase S-adenosylhomocysteine concentrations, which can inhibit methyltransferase activity and contribute to global DNA hypomethylation (Mandaviya et al., 2014). In this context, the concurrent identification of hyperhomocysteinemia and CDH7 hypomethylation as independent predictors in our model raises the possibility of a biological link between altered homocysteine metabolism and gene-specific methylation changes in CDH7 among patients with SVD. However, the present study was not designed to investigate causal mechanisms, and the metabolic and molecular pathways connecting homocysteine metabolism with cadherin-related vascular or neural functions remain to be elucidated.

Several limitations should be acknowledged. First, the EPIC array interrogates only a fraction of genomic CpG sites; broader epigenome-wide approaches such as whole-genome bisulfite sequencing and methylated DNA immunoprecipitation (Beck et al., 2022) may identify additional loci associated with SVD. Second, larger and longitudinal cohorts, as well as external validation, are needed to establish the robustness and temporal dynamics of *CDH7* hypomethylation as a biomarker. Third, although buffy coats are practical surrogate tissues, their epigenetic signals may not fully reflect brain-specific processes. Finally, blood samples were collected within days of acute ischemic stroke, and acute systemic responses could have influenced peripheral DNA methylation patterns.

In conclusion, this study suggests that *CDH7* promoter hypomethylation is associated with cerebral small vessel disease, with a possible preferential link to lacunar pathology. These findings highlight the potential relevance of epigenetic alterations in improving biological understanding of cerebral SVD, while emphasizing the need for further mechanistic, tissue-specific, and longitudinal studies before clinical or biomarker applications can be considered.

## Data Availability

The original contributions presented in the study are publicly available. This data can be found in the NCBI Gene Expression Omnibus (GEO) repository with the accession number GSE318387.
