# Supplementary material for: Promoter hypomethylation of CDH7: a novel epigenetic marker associated with cerebral small vessel disease
Source: Front Genet. 2026 Mar 12;17:1780415. doi: 10.3389/fgene.2026.1780415 (PMC13016587; doi:10.3389/fgene.2026.1780415)
Supplement: Supplementary file 2 [file Table2.docx]

Supplementary Material

**Supplementary Table 2.** Variance partitioning summary for the minfi and SeSAMe pipelines. Cell composition was estimated using RefFreeEWAS (K = 5) for the minfi pipeline and the EpiDISH blood reference for the SeSAMe pipeline. Hb, hemoglobin; SV, surrogate variable; Cell_Latent1-5, RefFreeEWAS latent components; Cell_B/CD4T/CD8T/NK/Neutrophils, EpiDISH-estimated leukocyte fractions; Mean values are variance proportions.

| Pipeline | Variable | Mean variance proportion |
| --- | --- | --- |
| minfi | primary_group | 0.001 |
|  | sex | 0.223 |
|  | Hb | 0.001 |
|  | Cell_Latent1 | 0.225 |
|  | Cell_Latent2 | 0.205 |
|  | Cell_Latent4 | 0.119 |
|  | Cell_Latent3 | 0.154 |
|  | Cell_Latent5 | 0.061 |
|  | SV1 | 0.002 |
|  | Residuals | 0.010 |
| SeSAMe | primary_group | 0.019 |
|  | sex | 0.043 |
|  | Hb | 0.032 |
|  | Cell_B | 0.036 |
|  | Cell_NK | 0.136 |
|  | Cell_CD4T | 0.072 |
|  | Cell_CD8T | 0.089 |
|  | Cell_Neutro | 0.221 |
|  | SV1 | 0.047 |
|  | Residuals | 0.306 |
