# Supplementary material for: Promoter hypomethylation of CDH7: a novel epigenetic marker associated with cerebral small vessel disease
Source: Front Genet. 2026 Mar 12;17:1780415. doi: 10.3389/fgene.2026.1780415 (PMC13016587; doi:10.3389/fgene.2026.1780415)
Supplement: Supplementary file 3 [file DataSheet1.pdf]

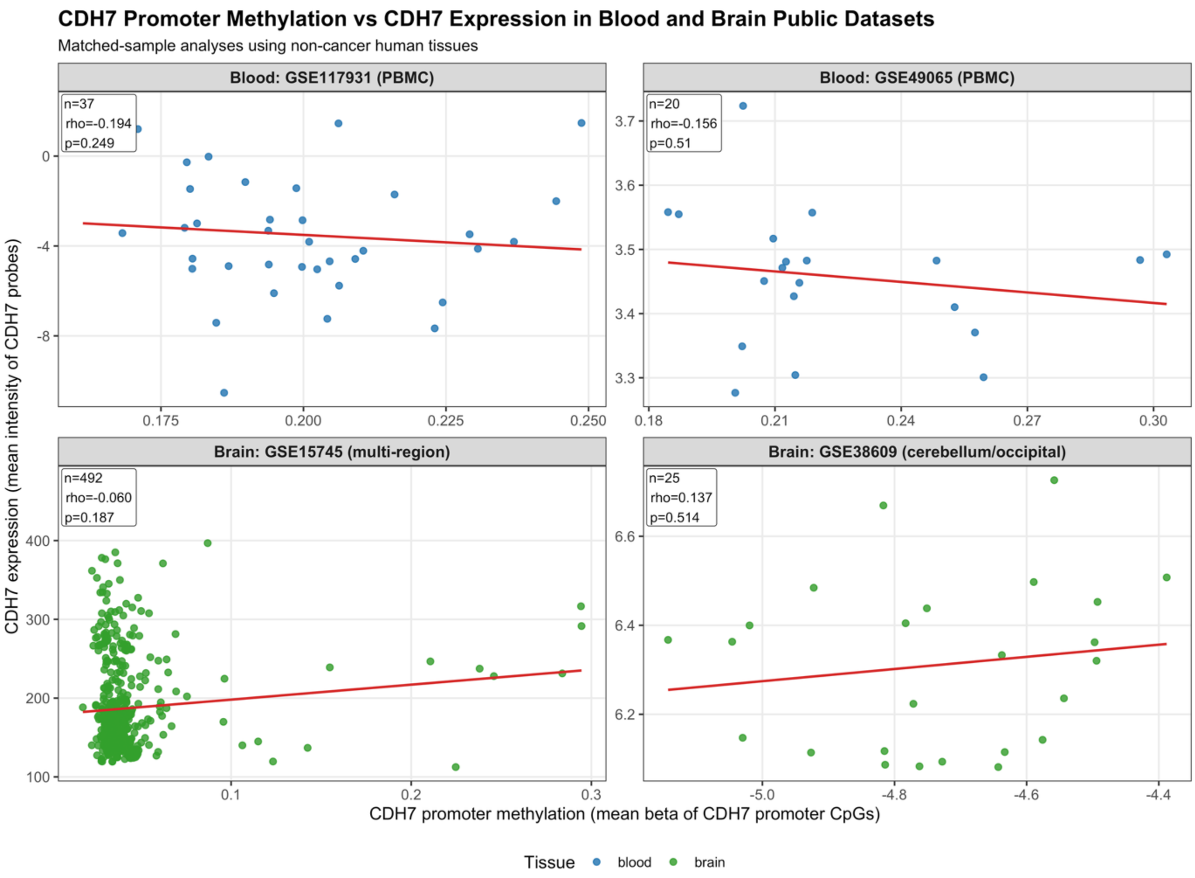

Supplementary Figure 1. Relationship between *CDH7* promoter methylation and *CDH7* expression in non-cancer human blood and brain datasets. Scatter plots show matched-sample associations between *CDH7* promoter methylation (x-axis; mean beta value across promoter-associated *CDH7* CpG probes) and *CDH7* expression (y-axis; mean signal across *CDH7* expression probes). Panels show GSE117931 PBMC (upper left), GSE49065 PBMC (upper right), GSE15745 brain tissue (bottom left), and GSE38609 brain tissue (bottom right). The red line indicates the fitted linear regression line.
