# Supplementary material for: Promoter hypomethylation of CDH7: a novel epigenetic marker associated with cerebral small vessel disease
Source: Front Genet. 2026 Mar 12;17:1780415. doi: 10.3389/fgene.2026.1780415 (PMC13016587; doi:10.3389/fgene.2026.1780415)
Supplement: Supplementary file 4 [file Table5.docx]

Supplementary Material

**Supplementary Table 5.** Number of missing values for each variable before imputation.

| Variables | Number of missing values |
| --- | --- |
| Homocysteine | 197 |
| High sensitivity C-reactive protein | 197 |
| Triglyceride | 46 |
| High density lipoprotein | 22 |
| Hemoglobin A1c | 22 |
| Low density lipoprotein | 17 |
| Total cholesterol | 11 |
| Creatinine | 8 |
| *CDH7* methylation | 2 |
| Total | 564 |

*CDH7*; cadherin-7.
