# Supplementary material for: Promoter hypomethylation of CDH7: a novel epigenetic marker associated with cerebral small vessel disease
Source: Front Genet. 2026 Mar 12;17:1780415. doi: 10.3389/fgene.2026.1780415 (PMC13016587; doi:10.3389/fgene.2026.1780415)
Supplement: Supplementary file 6 [file Table4.docx]

Supplementary Material

**Supplementary Table 4.** Comparison of pyrosequencing results between 16 all-SVD and 16 no-SVD patients.

| Gene name | Sample group | CpG 1 | CpG 2 | CpG 3 | CpG 4 | CpG 5 | Total | SD |
| --- | --- | --- | --- | --- | --- | --- | --- | --- |
| *CDH7* | Control | 23.4 | 23.5 | 22.4 | - | - | 23.1 | 7.37 |
|  | SVD | 25.6 | 26.5 | 25.3 | - | - | 25.8 | 10.5 |
| *ZNF234* | Control | 6.5 | 1.4 | 0.5 | 2.3 | 1.5 | 2.8 | 2.4 |
|  | SVD | 10 | 4.1 | 4.9 | 4.4 | 1.8 | 5.8 | 5.1 |

SVD, small vessel disease; *CDH7*; cadherin-7; SD, standard deviation.
